# Systematic analysis of phosphotyrosine antibodies recognizing single phosphorylated EPIYA-motifs in CagA of East Asian-type *Helicobacter pylori* strains

**DOI:** 10.1186/s12866-016-0820-6

**Published:** 2016-09-02

**Authors:** Judith Lind, Steffen Backert, Rebecca Hoffmann, Jutta Eichler, Yoshio Yamaoka, Guillermo I. Perez-Perez, Javier Torres, Heinrich Sticht, Nicole Tegtmeyer

**Affiliations:** 1 Department of Biology, Division of Microbiology, Friedrich Alexander University Erlangen-Nuremberg, Staudtstr. 5, D-91058 Erlangen, Germany; 2 Department of Chemistry and Pharmacy, Friedrich Alexander University Erlangen-Nuremberg, Schuhstraße 19, D-91052 Erlangen, Germany; 3 Department of Environmental and Preventive Medicine, Oita University Faculty of Medicine, Yufu, Japan; 4 Department of Medicine and Microbiology, New York University, Langone Medical Centre, New York, USA; 5 Unidad de Investigación en Enfermedades Infecciosas, Hospital de Pediatría del Instituto Mexicano del Seguro Social, Mexico City, México; 6 Bioinformatics, Institute for Biochemistry, Friedrich Alexander University Erlangen-Nuremberg, Fahrstrasse 17, D-91054 Erlangen, Germany

**Keywords:** c-Abl, c-Src, CagA, *cag*PAI, Dotblot, EPIYA motifs, Gastric cancer, *Helicobacter pylori*, Signaling, Type IV secretion, T4SS, Tyrosine kinases

## Abstract

**Background:**

Highly virulent strains of the gastric pathogen *Helicobacter pylori* encode a type IV secretion system (T4SS) that delivers the effector protein CagA into gastric epithelial cells. Translocated CagA undergoes tyrosine phosphorylation by members of the oncogenic c-Src and c-Abl host kinases at EPIYA-sequence motifs A, B and D in East Asian-type strains. These phosphorylated EPIYA-motifs serve as recognition sites for various SH2-domains containing human proteins, mediating interactions of CagA with host signaling factors to manipulate signal transduction pathways. Recognition of phospho-CagA is mainly based on the use of commercial pan-phosphotyrosine antibodies that were originally designed to detect phosphotyrosines in mammalian proteins. Specific anti-phospho-EPIYA antibodies for each of the three sites in CagA are not forthcoming.

**Results:**

This study was designed to systematically analyze the detection preferences of each phosphorylated East Asian CagA EPIYA-motif by pan-phosphotyrosine antibodies and to determine a minimal recognition sequence. We synthesized phospho- and non-phosphopeptides derived from each predominant EPIYA-site, and determined the recognition patterns by seven different pan-phosphotyrosine antibodies using Western blotting, and also investigated representative East Asian *H. pylori* isolates during infection. The results indicate that a total of only 9–11 amino acids containing the phosphorylated East Asian EPIYA-types are required and sufficient to detect the phosphopeptides with high specificity. However, the sequence recognition by the different antibodies was found to bear high variability. From the seven antibodies used, only four recognized all three phosphorylated EPIYA-motifs A, B and D similarly well. Two of the phosphotyrosine antibodies preferentially bound primarily to the phosphorylated motif A and D, while the seventh antibody failed to react with any of the phosphorylated EPIYA-motifs. Control experiments confirmed that none of the antibodies reacted with non-phospho-CagA peptides and in accordance were able to recognize phosphotyrosine proteins in human cells.

**Conclusions:**

The results of this study disclose the various binding preferences of commercial anti-phosphotyrosine antibodies for phospho-EPIYA-motifs, and are valuable in the application for further characterization of CagA phosphorylation events during infection with *H. pylori* and risk prediction for gastric disease development.

## Background

*Helicobacter pylori* is a human-specific pathogen colonizing the gastric mucosa of the stomach. About 50 % of the world's population carries this microbe, often causing asymptomatic gastritis in infected individuals, and more severe gastric diseases in up to 10–15 % of infected persons [1–4]. Although *H. pylori* infections are commonly associated with elevated inflammation parameters, the bacteria are not eliminated and can become persistent. Various mechanisms of host immune evasion were documented and *H. pylori* became a prime example of chronic bacterial infections. For example, it appears that *H. pylori* infection can efficiently reprogram dendritic cells toward a tolerogenic phenotype and induces regulatory T-cells with highly suppressive activity [5]. Further studies have indicated not only *H. pylori*’s remarkable capability to colonize individual persons for decades, but also that this bacterium has co-existed with modern humans for a very long time in history. Genetic studies showed that *H. pylori* spread together with its host during human migrations out of Africa about 58,000 years ago [6]. Due to this long time of co-evolution, there is growing evidence indicating that colonization by *H. pylori* could have also been advantageous for its human carriers supplying various benefits [3, 7]. For example, such advantages could include known protective effects of *H. pylori* against allergic and chronic inflammatory diseases [5]. In the modern world, however, infections with *H. pylori* can cause a serious burden of morbidity and mortality in the communities as a result of peptic ulceration, mucosa-associated lymphoid tissue (MALT) lymphoma and gastric cancer [1, 7, 8].

*H. pylori* strains are highly heterogeneous both in their DNA sequences and virulence. Dozens of bacterial genes have been described to control the pathogenesis of *H. pylori.* One of the best characterized virulence factors is the CagA protein encoded in the cytotoxin-associated genes (*cag*) pathogenicity island (PAI). The *cag*PAI encodes a type IV secretion system (T4SS), representing a needle-like pilus, which is induced upon contact with host cells [9–12]. CagA is translocated by this T4SS across the two bacterial and host cell membranes into the cytoplasm of target cells. CagA represents a prime example of tyrosine-phosphorylatable bacterial virulence factors [13–17]. Upon delivery, members of the c-Src [18, 19] and c-Abl [20, 21] host tyrosine kinase families were identified to phosphorylate CagA. Mass spectrometry and site-directed mutagenesis of CagA identified a set of Glu-Pro-Ile-Tyr-Ala (EPIYA) repeat motifs as phosphorylation sites [19, 22–26]. Four specific EPIYA-repeat motifs (named A, B, C and D) were described, primarily based on their relative position in CagA and flanking amino acid arrangements. These EPIYA-motifs were originally defined in 1993 by the group of Antonello Covacci [27] and reveal some diversity in adjoining sequences and even in the EPIYA-sites themselves [2, 28–30]. Although the majority of CagA proteins comprise three EPIYA-motifs, some isolates have less or additional EPIYA-copies in different combinations, due to recombination events between repeat sequences in the flanking DNA [29, 30]. The EPIYA-A and EPIYA-B sites are present in almost all CagA proteins worldwide. EPIYA-C is predominantly observed in isolates with Indo-European and African ancestry, while CagA of most East Asian *H. pylori* typically carry the EPIYA-D motif instead of EPIYA-C [28, 31–41]. Delivered CagA can interact with at least 20 host cell proteins, specifically in phosphorylation-dependent and phosphorylation-independent fashions, to hijack host cell signaling pathways involved in disease development [29]. A typical characteristic of AGS gastric epithelial cells infected with *cag*PAI-positive *H. pylori* is the “elongation” or “hummingbird” phenotype [13, 19, 22]. This in vitro phenotype likely mirrors numerous in vivo signaling activities that control host cell motility, invasive growth and metastasis of cancer cells [42, 43].

Phosphorylated CagA protein species present in AGS or MKN-28 cells infected with *H. pylori* carrying three EPIYA-motifs of Western (A, B, C) or East Asian (A, B, D) strains were analyzed by two-dimensional gel electrophoresis. In these studies it was demonstrated that only one or two tyrosines (but not three) can be phosphorylated per single CagA molecule [44, 45]. Interestingly, c-Src only phosphorylated EPIYA-C or EPIYA-D, while c-Abl phosphorylated EPIYA-A, EPIYA-B, EPIYA-C, and EPIYA-D [45]. Further analysis revealed that at least two phosphorylated EPIYA-motifs are required for triggering AGS cell elongation — the preferred combination in Western strains is EPIYA-A and EPIYA-C, either across two CagA molecules or simultaneously on one [45]. Site-directed mutagenesis further established a hierarchic phosphorylation model starting at EPIYA-C/D, followed by phosphorylation at EPIYA-A or EPIYA-B [45]. However, the observation that translocated or transfected CagA can be tyrosine-phosphorylated is mainly based on Western blotting using commercial pan-phosphotyrosine antibodies [13–17]. These antibodies were generated many years ago to identify phosphorylated tyrosine residues in mammalian proteins. A similar binding preference is displayed for mammalian phosphotyrosines by three of these α-phosphotyrosine antibodies, preferably with a leucine residue at position -1 and a proline at position +3 [46]. Interestingly, proline and leucine residues are not present at the corresponding position in CagA [29, 30, 47]. However, we have recently shown that at least three commercial phosphotyrosine-specific antibodies recognize the phosphorylated EPIYAs of many Western strains [48]. Nevertheless, systematic analyses on the specific recognition patterns of phosphorylated EPIYAs in East Asian CagAs by a large number of different antibodies were not yet reported. To address this important problem, we have utilized phospho- and non-phosphopeptides of each EPIYA-motif from East Asian strains and studied the recognition specificities by seven commercial α-phosphotyrosine antibodies. In addition, we performed infection experiments of AGS cells to investigate the recognition patterns of the phosphorylated CagAs upon translocation by East Asian *H. pylori* strains.

## Methods

### Phospho- and non-phospho CagA peptide synthesis

The C-STEPIYAKVNK, C-STEPI(pY)AKVNK, C-TEPI(pY)AKVN, C-EPI(pY)AKV and C-PI(pY)AK peptides were obtained from Biosyntan GmbH (Berlin/Germany) and the C-NTEPIYAQVNK (EPIYA-A), C-NTEPI(pY)AQVNK (phospho-EPIYA-A), C-PEEPIYAQVAK (EPIYA-B) and C-PEEPI(pY)AQVAK (phospho-EPIYA-B) sequences were synthesized by Jerini AG (Berlin/Germany). The C-SPEPIYATIDF (EPIYA-D) and C-SPEPI(pY)ATIDF (phospho-EPIYA-D) peptides were synthesized as described [49]. As α-phosphotyrosine antibodies usually recognize short phosphopeptides [40, 46, 50, 51], the indicated 11-mer peptides were selected to compare the three different EPIYA-motifs. Generally, 11-mer peptides are also used for immunizations to produce phospho-specific antibodies (Biogenes, Berlin/Germany). The peptides were dissolved in DMSO at a final concentration of 1 mg/mL and stored at -20 °C. Purification of all above EPIYA peptides was carried out by standard HPLC. The purity of each peptide as well as full-length synthesis was approved using mass spectrometry by Biosyntan GmbH and Jerini AG.

### *H. pylori* strains and mutagenesis

Seven *H. pylori* wild-type isolates from different Asian countries are *cag*PAI- and CagA-positive (Table 1). Isogenic Δ*cagA* and Δ*cagL* knockout mutants were generated according to standard procedures [52, 53]. All Helicobacters were raised on GC agar plates supplemented with nystatin (1 μg/mL), trimethoprim (5 μg/mL), vancomycin (10 μg/mL) and horse serum [54, 55]. The antibiotics were purchased from Sigma-Aldrich (St. Louis, MO/USA). The agar plates were cultivated for 2 days at 37 °C in anaerobic jars containing CampyGen packs (Oxoid, Wesel/Germany) generating an atmosphere of 85 % N_2_, 10 % CO_2_ and 5 % O_2_ [56].

**Table 1:** Characteristics of *H. pylori* strains and encoded CagA proteins used in this study

*H. pylori* strain	Origin	Pathology	CagA EPIYA-type	Protein sequence	Reference
Ind69	Indonesia	Gastric ulcer	ABD	LC007101	[97]
FD453	Malaysia	Functional dyspepsia	ABD	This study	[36]
Mand38	Myanmar	Gastritis (Antral predominant mild gastritis)	ABD	LC007102	This study
CH7	China	Pre-cancer surveillance study	ABD	This study	This study
TN2-GF4	Japan	Gastric ulcer	ABD	LC007103	[98]
2002-14	Mexico	Dyspepsia	ABD	JN390453	[99]
Shi470	Peruvian Amazon	Not evaluated directly (Non-atrophic gastritis assumed)	ABD	YP_001910294.1	[100]

### In vitro phosphorylation assay of CagA with Abl kinase

Wild-type CagA expressing *H. pylori* isolates TN2-GF4 and Mand38 (or isogenic Δ*cagA* mutants as control) were used for in vitro phosphorylation assays. Briefly, 10^10^ cells were lysed in 1 mL of kinase buffer as described previously and 30 μL of the *H. pylori* lysate were mixed with two units of human c-Abl tyrosine kinase in the presence of 1 μmol/L of ATP (NEB, Frankfurt/Germany) [57]. After incubation for 30 min at 30 °C, the reactions were stopped by heating the samples at 95 °C for 5 min [48].

### Dotblot analyses

Dot blot analyses were carried out according to standard protocols, using Immobilon-P membrane and the BioDot SF apparatus (Bio-Rad, Munich/Germany). Thirty μl of the in vitro kinase reaction products described above or 20 μg of each EPIYA peptide were mixed in 1 mL of transfer buffer (192 mM glycine, 25 mM Tris-HCl, 20 % methanol, 0.1 % SDS, pH 8.3). Subsequently, the samples were spotted onto the Immobilon-P membranes (Merck Millipore, Darmstadt/Germany). After drying, the Dotblots were incubated with the various antibodies as described below for the Western blots.

### Host cell culture and elongation phenotype quantification

AGS gastric adenocarcinoma epithelial cells (ATCC CRL-1730) were cultivated for two days on petri dishes in RPMI 1640 medium (Life Technologies GmbH, Darmstadt/Germany) [58]. Culture medium also contained 25 mM HEPES buffer and 10 % fetal bovine serum (FBS; Biochrom, Berlin/Germany), which was heat-inactivated [59, 60]. Before infection, AGS cells were washed with PBS (phosphate-buffered saline) and incubated with serum-depleted fresh medium for 12 h. Infection with *H. pylori* was commonly performed for 6 h at a multiplicity of infection (MOI) of 50. The cells were then harvested in ice-cold PBS in the presence of 1 mmol/L Na_3_VO_4_ (Sigma-Aldrich). In each experiment the number of elongated AGS cells was quantified in three different 0.25-mm^2^ fields using a phase contrast microscope (Olympus IX50). All experiments were done in triplicates and the results were analyzed statistically as described below.

### SDS-PAGE and Western blotting

Infected AGS cells were harvested by adding hot SDS loading buffer to the culture plates. Then, the samples were collected, incubated for 5 min at 95 °C, loaded on 6 % SDS-PAGE gels and blotted onto Immobilon-P membranes. After blocking the membranes in TBST buffer with 5 % skim milk or with 3 % bovine serum albumin (BSA) for 1 hour at room temperature, they were incubated with rabbit polyclonal α-CagA antibody (Austral Biologicals, San Ramon, CA/USA) or with the seven commercial α-phosphotyrosine antibodies (Table 2). Details on dilution and buffer conditions for each of these antibodies have been provided recently [48]. Horseradish peroxidase-labelled anti-mouse or anti-rabbit polyvalent goat immunoglobulins were used as secondary antibodies [61, 62]. Detection of phosphorylated and non-phosphorylated CagA proteins was performed with the ECL Plus chemoluminescence Western blot kit (GE Healthcare, Freiburg/Germany) [63, 64].

**Table 2:** Recognition of EPIYA-phosphopeptides and phosphorylated CagA proteins by commercial α-phosphotyrosine antibodies

Phospho-antibody name	Company name	Recognition of phosphopeptides			Relative phosphorylation signal intensity of translocated CagA by *H. pylori* strains						
		EPIYA-A	EPIYA-B	EPIYA-D	Ind69	F453	Mand38	CH7	TN2-GF4	2002-14	Shi470
α-PY-99	Santa Cruz Biotech	+++	+++	+++	+++	+++	+++	+++	+++	+++	+++
α-PY-20 (BD)	BD Biosciences	+++	+++	+++	+/-	++	+/-	+++	+++	+++	+++
α-PY-20 (SC)	Santa Cruz Biotech	+++	+++	+++	+/-	++	+/-	++	+++	+++	+++
α-PY-100	Cell Signaling	+++	+	+++	-	+	-	+	++	++	++
α-PY-69	BD Biosciences	+++	++	++	+	++	++	++	+++	+++	++
α-PY-102	Cell Signaling	+++	-	++	+/-	+	+/-	+	++	++	+++
α-PY-350	Santa Cruz Biotech	-	-	-	-	-	-	-	-	-	-

*Abbreviations: PY* (phosphotyrosine), *EPIYA* motif (glutamic acid-proline-isoleucine-tyrosine-alanine phosphorylation motif in CagA), *Antibody recognition:* +++ (strong signal); ++ (moderate signal); + (weak signal); - (no signal)

### Quantitation of signals in Western blot and Dotblot

Quantification of band or spot intensities on immunoblots was performed using the Chemicdoc imaging system (Bio-Rad) and indicated the percentage of phosphorylation per sample [65]. As represented in the corresponding figures the strongest spot on each Dotblot was set at 100 %.

### Statistical examination

The Student *t*-test was performed using SigmaPlot statistical software (version 13.0) to evaluate all data. All error bars shown in figures and those quoted following the +/- signs represent standard deviations.

## Results

### Short CagA-derived phosphopeptides are sufficient for recognition by α-phosphotyrosine antibodies

**Fig. 1:**
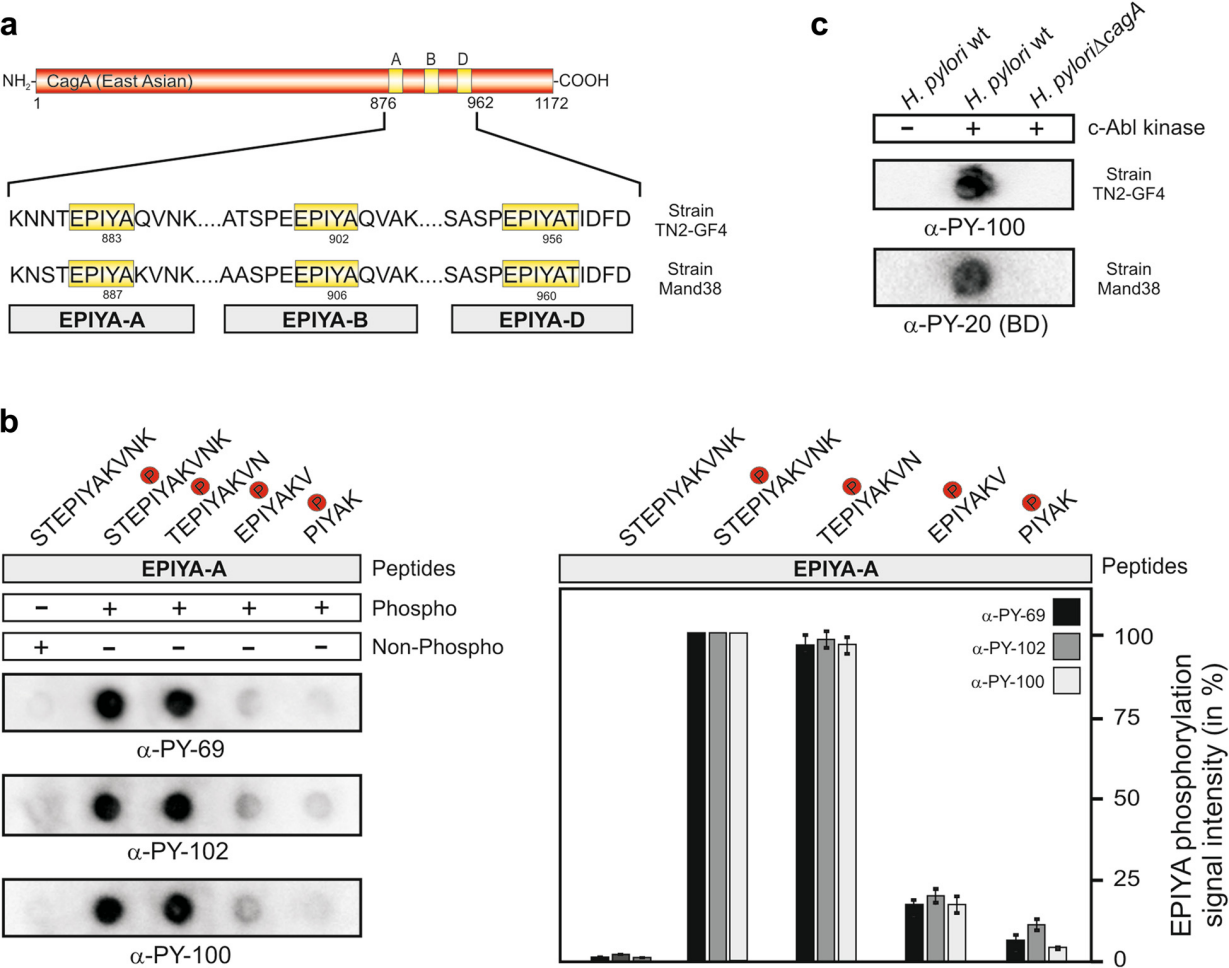
Detection of short EPIYA-phosphopeptides from two *H. pylori* East Asian CagAs by α-phosphotyrosine antibodies. **a** East Asian CagA proteins of *H. pylori*, as shown here for the strains TN2-GF4 and Mand38, primarily carry the EPIYA-A, EPIYA-B and EPIYA-D segments (Table 1). These motifs can be phosphorylated by c-Abl and c-Src host kinases. **b** Various truncated EPIYA-A motif-derived phospho- and non-phosphopeptides from strain Mand38 were generated and analysed with the Dotblot method. These peptides were immobilized on PVDF membranes and probed with the indicated phosphotyrosine antibodies. On the right site, spot intensities derived from three independent experiments of the detected spots are quantified. The intensities of the signals were measured densitometrically by the Chemidoc imager and display the percentage of phosphorylation signal per sample. For every Dotblot the strongest spot was set to 100 % for each of the different α-phosphotyrosine antibodies as indicated. The resulting data indicate that 9-mer and 11-mer phosphopeptides are sufficient to reveal solid recognition by the antibodies. **c** Products of in vitro kinase reactions of c-Abl with bacterial lysates (from *H. pylori* wild-type strain TN2-GF4, Mand38 and isogenic Δ*cagA* mutant) were employed for control Dotblot experiments using antibodies α-PY-100 and α-PY20

The East Asian CagA proteins typically harbor three phosphorylatable sequence motifs, called EPIYA-A, -B and -D, as indicated for the *H. pylori* strains TN2-GF4 and Mand38 (Fig. 1a). It was previously shown that short mammalian derived phosphopeptides can be recognized by commercial α-phosphotyrosine antibodies and in various studies only five amino acid residues were sometimes sufficient for strong binding [40, 46, 50, 51]. We therefore performed a systematic analysis on the recognition capacities of various phosphorylated East Asian CagA peptides by these α-phosphotyrosine antibodies. We first synthesized a collection of peptides derived from the EPIYA-A site of Mand38 displaying the phosphotyrosine in the center plus five, four, three or two flanking amino acid residues on each side, including the PIYAK (5-mer), EPIYAKV (7-mer), TEPIYAKVN (9-mer) and STEPIYAKVNK (11-mer) sequences as shown (Fig. 1b, top). Using the Dotblot technique, twenty μg of each EPIYA-peptide were immobilized on PVDF membranes per spot and subsequently analyzed with the α-phosphotyrosine antibodies α-PY-69, α-PY-102 and PY-100, respectively. All three antibodies were able to recognize 11-mers and 9-mers with comparable strong intensity. However, the recognition of 7-mer and 5-mer peptides was substantially reduced (Fig. 1b). As control experiment, an 11-mer peptide of the equivalent non-phospho-EPIYA motif did not result in any phospho-signal (Fig. 1b). Additional Dotblot experiments using *H. pylori* lysates of TN2-GF4 and Mand38 confirmed the presence of phospho-CagA when co-incubated with Abl in in vitro kinase reactions (Fig. 1c). In this way, we could also confirm that the α-phosphotyrosine antibodies do not cross-react with non-phosphorylated East Asian CagA forms in control reactions in the absence of Abl kinase (Fig. 1c). Taken together, these results validate the Dotblot method useful for studying CagA phosphorylation sites and demonstrate that α-phosphotyrosine antibodies can profoundly recognize East Asian 9-mer and 11-mer phospho-EPIYA sequences.

### Recognition of East Asian EPIYA-A, -B and -D phosphopeptides by α-phosphotyrosine antibodies

**Fig. 2:**
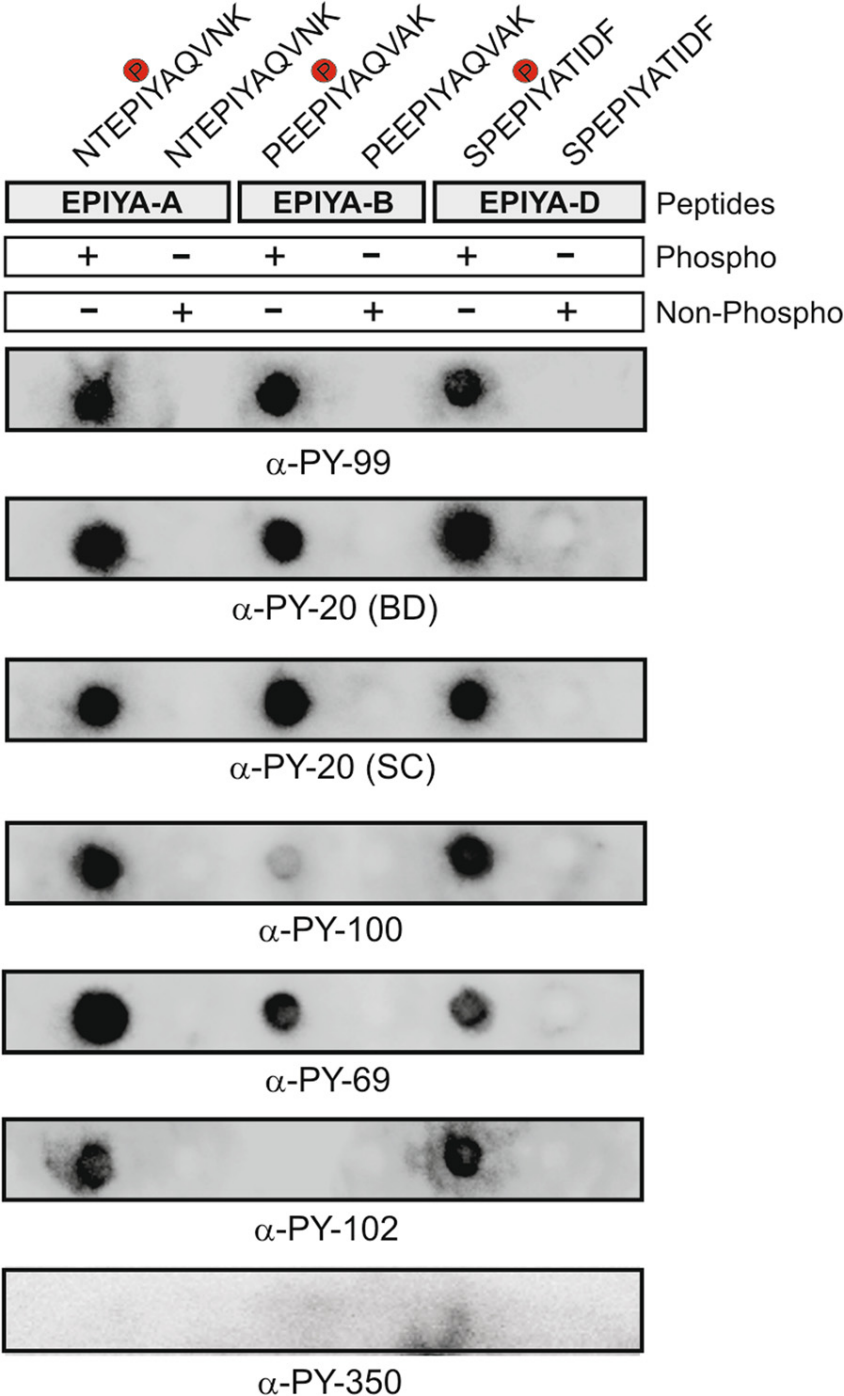
Different recognition capacities of synthetic 11-mer CagA phosphopeptides by seven commercially available α-phosphotyrosine antibodies. The indicated phospho- and non-phospho peptides derived from single East-Asian EPIYA-motifs A, B and D of *H. pylori* wild-type strain TN2-GF4 were analyzed using the Dotblot method. The seven designated phosphotyrosine-specific antibodies were used as previously described [48]

As next, we synthesized 11-mer phospho- and non-phosphopeptides of EPIYA-A (NTEPIYAQVNK), EPIYA-B (PEEPIYAQVAK) and EPIYA-D (SPEPIYATIDF) motifs of strain TN2-GF4 as indicated in Fig. 2 (top). Resulting Dotblots were probed with a collection of seven commercial α-phosphotyrosine antibodies in order to test for their binding specificity of individual EPIYA-motifs. The control blots show that the corresponding non-phospho CagA peptides did not reveal any signal, thus confirming that none of the antibodies produced false-positive results (Fig. 2). The majority of the antibodies [α-PY-20 (BD), α-PY-20 (SC), α-PY-69, α-PY-99, α-PY-100, α-PY-102] primarily recognized the East Asian-type EPIYA-A and EPIYA-D phosphopeptides. Reaction with the EPIYA-B phosphopeptide revealed a mixed recognition capacity, where the antibodies α-PY-100 resulted in only low detection and α-PY-102 was unable to detect the EPIYA-B phosphopeptide at all. The antibodies α-PY-99, α-PY-20 (BD) and α-PY-20 (SC) were able to detect the phosphopeptides derived from all three EPIYA-motifs (A, B and D), while the antibody PY-100 and PY-102 preferentially reacted with the EPIYA phosphopeptides A and D (Fig. 2). The only exception was antibody PY-350, which not produce a signal with any of the EPIYA phosphopeptides. Increasing the amount of bound peptide up to five-fold or doubling the amount of PY-350 antibody failed to yield any signal with the EPIYA phosphopeptides (Fig. 2), but the antibody successfully detected phosphorylated host cell proteins, thus confirming its general functionality (Fig. 6). These results suggest that six of the seven commercial α-phosphotyrosine antibodies recognize the various East Asian CagA phospho-EPIYA motifs to different extent.

### Comparison of East Asian- and Western-type EPIYA peptide recognition by α-phosphotyrosine antibodies

**Fig. 3:**
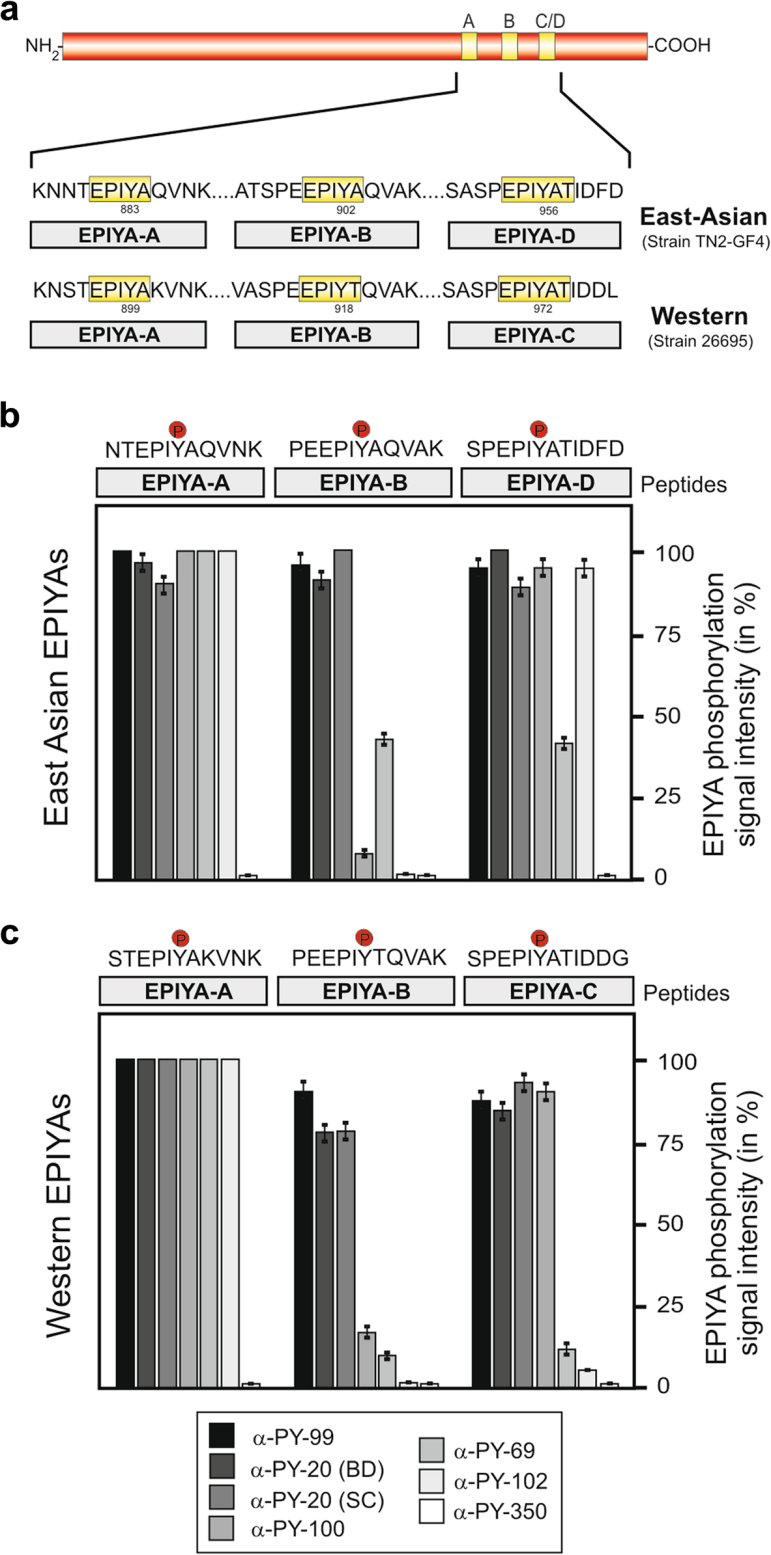
Comparison of phospho-signal intensities for East Asian- and Western-type EPIYA motifs by seven commercial α-phosphotyrosine antibodies. **a** Schematic presentation of CagA EPIYA-motifs, comprising either the EPIYA-A, EPIYA-B and EPIYA-C motif in Western-type *H. pylori* strains as here shown for the strain 26695 or the East Asian-type CagA EPIYA-motifs in which the EPIYA-C region is replaced by EPIYA-D as found in strain TN2-GF4. **b** Quantified spot intensities of East Asian-type derived EPIYA-motifs A, B and D were probed with seven commercially available phosphotyrosine antibodies as indicated. The Chemidoc imager was used to measure densitometrically the percentage of phosphorylation of each sample. The data are representative from three independent experiments, where the strongest spot on each Dotblot was set at 100 %. **c** Quantification of spot intensities of corresponding phosphotyrosine peptides derived from Western-type EPIYA-motifs A, B and C. These data were taken from our previous study [48]

We have recently reported the recognition patterns of 11-mer Western-type phospho-EPIYA motifs by α-phosphotyrosine antibodies [48]. These Western-type EPIYA-motifs differ in a few amino acids from the East Asian counterparts (Fig. 3a). In order to investigate if alteration in some defined flanking amino acid residues may change the antibody binding patterns, we compared the recognition capabilities of East Asian- and Western-type EPIYA peptides by the various α-phosphotyrosine antibodies. The EPIYA-A motif was similarly well recognized by all six above mentioned antibodies, regardless if the phosphopeptide derived from Western (26695) or East Asian (TN2-GF4) *H. pylori* strains (Fig. 3b-c). The same was true for the phosphopeptides derived from the EPIYA-B motif, although one antibody (α-PY69) exhibited higher detection ability for the East-Asian phosphopeptide compared to the Western counterpart. In this context it is interesting to note that the EPIYA-motifs differ only by a single amino acid exchange, namely T → A at the +1 position behind the phosphotyrosine. This exchange was shown previously to affect the interaction of CagA with the SH2-domain of PI3-kinase [66]. The present data shows that the identity of the residue at position +1 does not only affect SH2-domain binding, but also the binding by some antibodies like α-PY69. In addition, the phospho-EPIYA-C/D derived peptides are similarly well recognized by four antibodies [α-PY-20 (BD), α-PY-20 (SC), α-PY-99, α-PY-100]. However, the antibodies α-PY69 and α-PY102 exhibited a much stronger binding of the East Asian-type EPIYA-D phosphopeptide compared to the Western-type EPIYA-C motif. However, the two peptides only differ by the amino acids at the +5 and +6 position (DG → FD), between the Western-type and the East Asian EPIYA-motif. This finding suggests that also amino acids, which are not located immediately adjacent to the phosphotyrosine residue, can critically affect the binding properties of some antibodies. In summary, it becomes apparent that the use of these antibodies results in some differences regarding the recognition capability not only for the EPIYA-motif derived from phosphopeptides of Western-type strains, but also of the three phospho-EPIYA-motifs A, B and D present in East Asian isolates.

### Sequence comparison of CagA proteins from East Asian strains

**Fig. 4:**
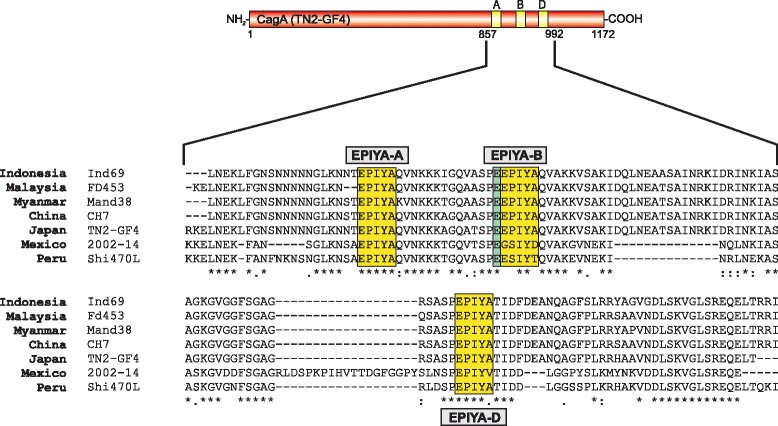
Alignment of EPIYA-motif sequences in CagA proteins derived from seven geographically different *H. pylori* strains. All chosen strains carry the typical East Asian CagA protein with the carboxy-terminal EPIYA-A, EPIYA-B and EPIYA-D phosphorylation sites. Host kinases such as c-Src and c-Abl were shown to target and phosphorylate the tyrosine residue in these motifs. The specific EPIYA-segments A, B and D are indicated with yellow shading and exhibit variations in their flanking regions depending on their geographical origins. A special attribute is present in the EPIYA-B motif by the presence of a negatively charged glutamate residue in the -4 position (shaded with green). This residue is highly conserved in EPIYA-B among the different *H. pylori* strains and might lead to alterations of the binding capacity of phosphotyrosine antibodies as discussed in the text. The CagA protein sequences were obtained either from databases or sequenced during this study (Table 1). Sequence alignment was done using the ClustalW2 program (http://www.ebi.ac.uk/Tools/msa/clustalw2/)

Having clarified the detection capacity of short East Asian EPIYA peptides by seven α-phosphotyrosine antibodies, we next aimed to look at full-length CagA proteins in corresponding *H. pylori* strains. Seven different isolates were selected from different countries including Indonesia, Malaysia, Myanmar, China, Japan, Mexico and Peru. All of them encode the tripartite East Asian-type EPIYA-A, B and D motifs in CagA, although the strains comprise differences in the associated gastric diseases (Table 1). Their pathology was associated with diverse symptoms ranging from mild metabolic disorders such as gastritis to even ulcer and even gastric cancer. By aligning and comparing the CagA sequences comprising the EPIYA-regions A, B and D, the presence of all three motifs could be confirmed, while a few differences in their flanking amino acid sequences were detected (Fig. 4). In addition, all strains carry a highly conserved glutamate residue at the -4 position of the EPIYA-B motif, but not EPIYA-A or EPIYA-D, which might affect antibody recognition after tyrosine phosphorylation [48]. Finally, we noted extensive variations in the less conserved EPIYA-A motif, which might also influence antibody binding as discussed below.

### Phospho-CagA protein patterns during infection with East Asian strains

**Fig. 5:**
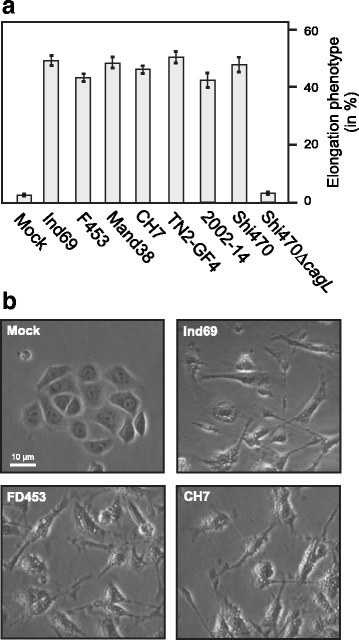
Phase contrast microscopy of AGS cells and quantification of the elongation phenotype during infection with various East Asian *H. pylori* strains. **a** Different indicated CagA-expressing East Asian *H. pylori* strains were co-incubated with AGS cells for 6 h. Afterwards, the number of elongated cells was quantified in triplicates. **b** AGS cells infected with selected *H. pylori* strains were analyzed by phase contrast microscopy

**Fig. 6:**
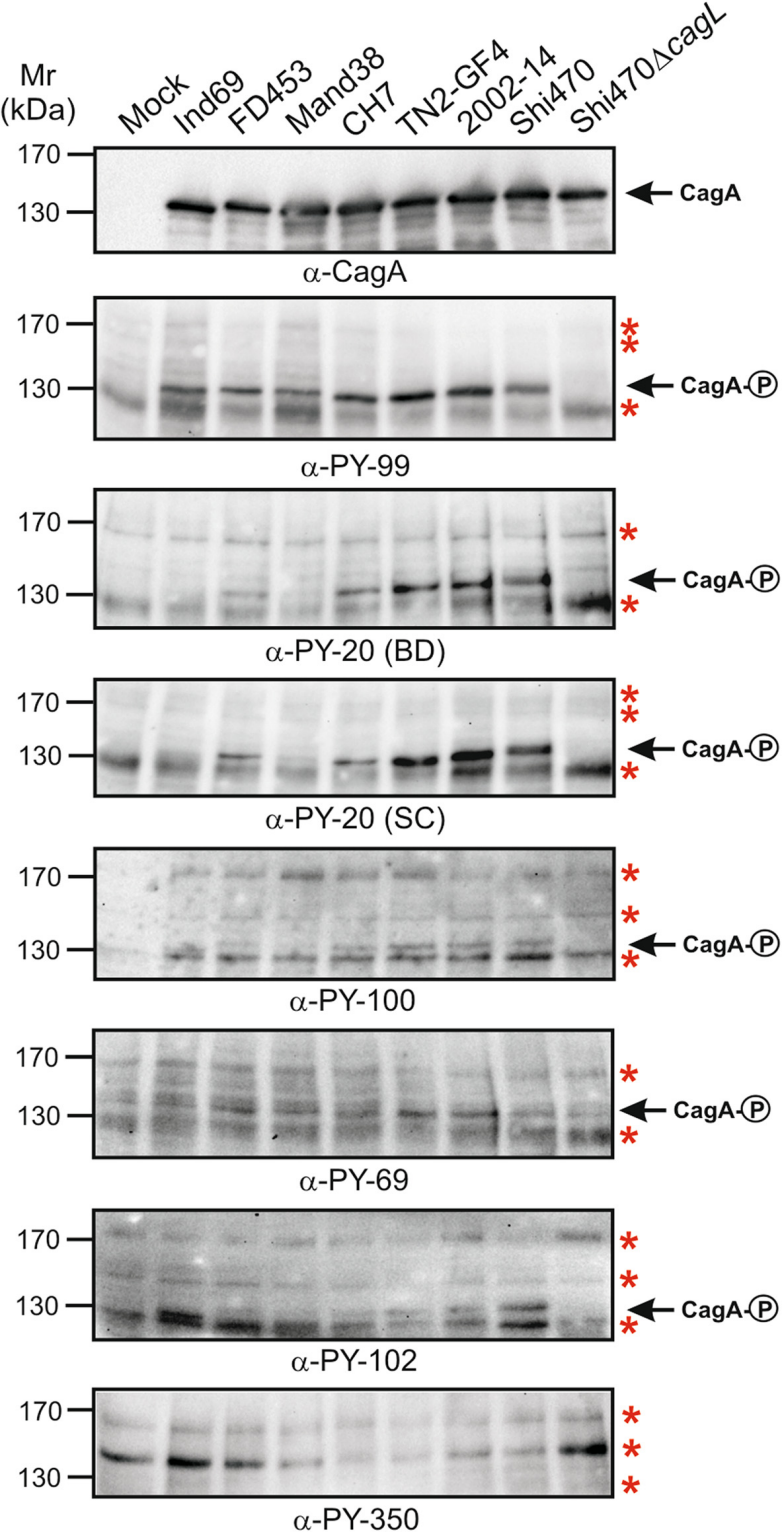
CagA phosphorylation at EPIYA-motifs during *H. pylori* infection of AGS cells was investigated using seven different α-phosphotyrosine antibodies. Seven different CagA-expressing East Asian-type *H. pylori* strains as well as the T4SS-inactive negative control (Shi470*ΔcagL*) were used for infection studies on AGS cells. The infection was monitored over 6 h and the samples shown in Fig. 5 were harvested after photographing. Tyrosine phosphorylation of EPIYA-motifs in CagA of the seven different strains was analyzed with the indicated α-phosphotyrosine antibodies as previously described [48]. Presence of equal amounts of CagA from each sample was approved using a monoclonal α-CagA antibody. The ~120-170 kDa section of the gels is shown. Arrows indicate the phospho-CagA bands, while red asterisks mark bands of various tyrosine-phosphorylated host cell proteins

To study antibody capabilities of phospho-CagA recognition during infection, we co-incubated AGS cells with the seven aforementioned East Asian *H. pylori* for 6 hours. We first monitored the elongation phenotype of AGS cells as this indicates successful CagA delivery and phosphorylation [66–68]. The elongation phenotype of AGS cells was found in around 50 % of cells after infection, confirming that an efficient amount of phospho-CagA should be present in the cells (Fig. 5a and b). Subsequently protein lysates derived from the infected AGS cells were prepared and tested with the different α-phosphotyrosine antibodies. To ensure that comparable amounts of CagA protein are present in all lanes, the samples were first incubated with a monoclonal α-CagA antibody which is able to recognize phosphorylated and non-phosphorylated CagA (Fig. 6, top). The band sizes varied between 130–150 kDa dependent on the different CagAs of the diverse strains used (Table 1). In addition, we infected with a Δ*cagL* mutant *H. pylori* strain as control, which has a T4SS defect for translocation and phosphorylation of CagA (Fig. 6, arrows). In the next step, the protein lysates were probed with the seven different α-phosphotyrosine antibodies. All antibodies were able to react with host cell proteins (Fig. 6, asterisks), and with the exception of α-PY-350, all of them were also able to recognize phospho-CagA (Fig. 6, arrows). Results of phospho-CagA detection of three independent experiments are summarized in Table 2. The antibody PY-99 was able to react strongly with the phospho-CagA of all seven strains and resulted only in little host phosphoprotein background in the 125–170 kDa region (Fig. 6). This confirms the presence of phospho-CagA in a sufficient and detectable manner indicating successful infection, which is in accordance with the detected elongation phenotype of AGS cells. Antibodies α-PY20-BD, α-PY20-SC, PY69 and α-PY102 recognized phospho-CagAs of all seven used *H. pylori* strains, while α-﻿PY100 was unable to react with CagA of strains Ind69 and Mand38, although it reacted with all three phospho-EPIYAs in the above mentioned Dotblot experiments. Strong bands were detected for phospho-CagA for three of the strains (TN2-GF4, 2002-14 and Shi470) with six of the seven used antibodies, while for the other strains (Ind69, F453, Mand38 and CH7) Western blotting revealed quite mixed results (Fig. 6). The phospho-CagA patterns were found to be not identical even among those antibodies that equally well recognized the samples of strains TN2-GF4, 2002-14 and Shi470. Phospho-CagA from strain Mand38 resulted in strong signals using α-PY69, but reacted only weakly with α-PY20. Again, one of the antibodies (α-PY350) was unable to react with any of the phospho-CagAs of the seven used *H. pylori* strains, corresponding to the results found for the used EPIYA phosphopeptides in Dotblot experiments (Fig. 2). Nevertheless, α-PY350 did react with host phosphoproteins, verifying the functionality of the antibody (Fig. 6, bottom).

### Recognition patterns of phospho-EPIYAs are diversely influenced by length and sequence

By applying the microarray technology, libraries of mammalian phosphoproteins were screened to define the antibody binding characteristics of the phosphotyrosine antibodies α-PY20 and α-PY100 [46]. Features of the recognized sequences by these antibodies revealed the differences accounting for the different binding capacity of the phosphopeptide EPIYA-B. By comparing the binding capacity of α-PY100 and α-PY20, it becomes obvious that the EPIYA-B motif is recognized with low intensity by α-PY100. This is in contrast to the two other motifs EPIYA-A and D (Fig. 2, Table 2). Remarkably, the EPIYA-B phosphopeptide carries a highly conserved glutamate residue in the -4 position in all of the used strains of this study (Fig. 4, shaded with green). This negatively charged glutamate residue might negatively affect the binding of α-PY100 but not of α-PY20 as indicated in the microarray data of Tinti and co-workers [46]. Accordingly, the differences in binding capacities found for the East Asian-type phospho EPIYA-B motif for the two antibodies correspond to the results of mammalian phosphoproteins and the negative charged amino acid at this position. Infection experiments also revealed a better detection of phosphorylated CagA by α-PY20 than α-PY100 which might arise from differences in the EPIYA-A sequences of the used strains (Fig. 6 and Table 2). The EPIYA-A stretch usually carries more variations in its vicinity than the EPIYA-B and D motif which contain more conserved regions (Fig. 4). Two of the *H. pylori* strains, Ind69 and Mand38, are not recognized at all by α-PY100 (Fig. 6), which however cannot be directly linked to any amino acids flanking the EPIYA-motifs (Fig. 4). This indicates that additional characteristics, like the accessibility of the motifs within the intact protein, further contribute to the binding capacity of α-PY100.

## Discussion

Posttranslational modification of proteins by kinases regulates various cell signaling processes. Phosphorylation of specific threonine, serine and histidine amino acid residues appears both in eukaryotes and prokaryotes, while tyrosine phosphorylation is considered to be more common in higher organisms [69, 70]. Phosphotyrosines represent a recognition site in higher eukaryotes because these motifs can recruit cellular binding partners that contain SH2 (Src homology 2) or PTB (phosphotyrosine binding) domains, and thereby target and subvert downstream signal transduction pathways [47]. In fact, genes encoding typical tyrosine kinases as known from eukaryotes have been only found in a very small number of bacterial species [71]. Instead, various (but not all) bacteria contain a group of atypical BY kinases (for *﻿B*acterial t*Y*rosine kinases) [72, 73]. SH2- and PTB-domain containing proteins are commonly missing in bacteria. Thus, tyrosine phosphorylation has a different role in bacteria and eukaryotes, respectively. However, various reports indicated that a series of effector proteins from pathogenic bacteria and viruses can be tyrosine-phosphorylated by host kinases upon delivery into mammalian target cells [29, 30, 47, 74]. This virulence mechanism is described for well-known microbial effector proteins including AnkA (*Anaplasma phagocytophilum, Ehrlichia chaffeensis*)*,* BepD-F (*Bartonella henselae*), TARP (*Chlamydia trachomatis*)*,* Tir (enteropathogenic *Escherichia coli*), CagA (*Helicobacter pylori*), LspA1/2 (*Haemophilus ducreyi*) and A36R (vaccinia virus) [74–82]. Interestingly, the phosphorylated tyrosines and some flanking amino acids in these microbial effectors, like their mammalian counterparts, serve as recognition motifs for host signaling proteins. These factors specifically recruit multiple host cell binding partners that harbor SH2 domains (but not PTB domains), thereby targeting and subverting mammalian signal transduction cascades in a manner supporting the infection cycle [74].

The impact of the well-known virulence factor CagA together with its EPIYA-motifs has been noted long time ago [2, 19, 22–31, 83–86]. Different gastrointestinal diseases have so far been found to be associated with sequence variation in the EPIYA-region of different *H. pylori* strains [87, 88] before these sites were recognized as tyrosine phosphorylation targets [84]. Since then, intensive studies have been brought forward to identify the required host cell kinases [29]. In mammalian genomes about 90 protein tyrosine kinase genes have been detected [70, 89]. Their mammalian substrates are phosphorylated with different specificity depending on amino acid sequences next to the targeted tyrosine residue [90]. The EPIYA-motifs in CagA primarily exhibit the small amino acid alanine at the +1 position and isoleucine at the -1 position, which is analogous to the EEIYG/E phosphorylation consensus motif of the host kinase c-Src [18]. In fact, members of the c-Src and c-Abl family kinases have been found to facilitate CagA phosphorylation in vitro and in vivo [18–21, 45, 91]. However, lack of standardized commercial EPIYA-specific phospho-antibodies and the lack of knowledge which phospho-EPIYAs are recognized by the set of available α-phosphotyrosine antibodies have made the progress in this research area vulnerable. So far, reports about systematic studies of which phosphotyrosine residues in the three EPIYA-sites are detected by these multiple antibodies are widely missing and only analyzed for some Western *H. pylori* strains [48]. Thus, despite many years of research, CagA phosphorylation patterns in clinical isolates have not been standardized to allow a thorough and precise model for this important signaling event. In the present study, we investigated for the first time East Asian-type CagA EPIYA-motifs A, B and D with respect to their recognition specificity by seven commercially available α-phosphotyrosine antibodies. Using this approach, we obtained significant recognition patterns for the various phosphorylated EPIYAs. The results of these studies are compared to their Western counterparts and allow valuable conclusions about the effectiveness of these antibodies in research and give new insights for upcoming work on CagA phosphorylation and associated signaling events.

The set of α-phosphotyrosine antibodies typically recognizes short amino acid stretches containing the phosphorylated tyrosine residue and were originally established for mammalian proteins and synthetic phosphopeptides [40, 46, 50, 51]. To study the recognition capabilities by seven commercial antibodies for the CagA EPIYA-motifs, we therefore proposed that corresponding phosphopeptides would be useful as shown previously for Western-type CagA EPIYAs [48]. In East Asian CagAs it was found that 9-mers and 11-mers of EPIYA-phosphopeptides are required and already sufficient for strong antibody binding. In addition, all three 11-mer phospho-EPIYA peptides (A, B and D) were recognized by three α-phosphotyrosine antibodies (α-PY69, α-PY-102 and α-PY-100) with similar and very strong signals, which confirm that peptides derived from bacterial effector proteins in addition to mammalian peptides can be detected with this approach. Generally, this also nicely reflects the pronounced recognition of phospho-CagA in cell lysates produced after infection with seven different *H. pylori* strains (Table 2). The phospho-EPIYA peptides A and D were preferentially also detected by another antibody (α-PY100) and in part gave rise to acceptable phospho-CagA patterns by Western blotting of proteins from infected cells. The antibody α-PY102 strongly recognized phospho-EPIYA peptide A and phospho-EPIYA peptide D, but reacted only with threeof eight phospho-CagAs in infected cells. The antibody α-PY69 also recognized phospho-EPIYA-A preferentially and to a lesser extent also EPIYA-B and D. In addition, it resulted in proper detection of phospho-CagA in all seven *H. pylori* strains during infection experiments. However, it also strongly reacted with host cell proteins in the 125–140 kDa range and is therefore not useful for studying CagA phosphorylation during infection. Noteworthy, the antibodies α-PY99, α-PY20-BD, α-PY20-SC, α-PY100 and α-PY102 did not react with AGS host cell proteins in the 130–150 kDa range. Similar to results obtained with Western-type *H. pylori* strains [48], the use of up to five α-phosphotyrosine antibodies for studies of infection by Asian-type *H. pylori* (α-PY99, α-PY20-BD and α-PY20-SC, and if needed, also α-PY100 and α-PY69) can be recommended to clarify EPIYA phosphorylation, as they are able to recognise a wide array of different phospho-CagAs.

In mammals, studies of phosphotyrosine-mediated protein-protein interactions are mainly based on the use of mass spectrometry and α-phosphotyrosine antibodies [69]. By using microarrays of spotted human phosphopeptides, the substrate binding specificity of two widely used α-phosphotyrosine antibodies, α-PY20 and α-PY100, was characterized [46]. The studies of Tinti and co-workers demonstrated that the antibodies share a similar phosphotyrosine recognition capability and comprise specific binding preferences depending on some neighboring amino acids [46]. Although leucine residues are favored at position -1 and proline at position +3, their binding preference remains rather broad [46]. Furthermore, it was found that the presence of a negatively charged residue (e.g. glutamate) at the position -4 specifically affects the interaction with α-PY100, but not with α-PY20 [46]. A highly conserved glutamate residue at the position -4 in EPIYA-B is present in CagAs from different *H. pylori* isolates (Fig. 4). By analyzing the results of Table 2 from the current study on East Asian-type strains together with the investigation on the Western-type *H. pylori* isolates [48], it becomes evident that additional features affect α-PY100 binding preference, as demonstrated by the low recognition of phospho-CagA from strains Ind69, F453, Mand38 and CH7. Because these *H. pylori* strains differ at some sequence positions in the close area of the EPIYA-A motif, a clear correlation regarding antibody binding with a single sequence position still remains elusive. We propose that the secondary structure of the EPIYA-motif and its surrounding might also contribute to the binding specificity by the α-phosphotyrosine antibodies.

Previous infection studies reported clear results regarding the phosphorylation of CagA EPIYA motifs [13, 14, 16, 19, 92–94], however, most of them used α-PY20 or α-PY99 phosphotyrosine antibodies, which allows detection of a multitude of Western and East Asian CagAs and is correlating well with the obtained results in the current study. Moreover, most reports were not specifically aiming for detection of specific EPIYA motifs, but rather CagA tyrosine phosphorylation in general. Only a few studies were aiming on the recognition of specific motifs of the investigated strains and prepared EPIYA-site specific tyrosine antibodies [11, 25, 26]. However, studies on tyrosine phosphorylation of different *H. pylori* strains might be influenced by the choice of the phosphotyrosine antibody. The study of Naito et al. [33] or Highashi et al. [23] utilized the 4G10 anti-phosphotyrosine for their studies on CagA tyrosine phosphorylation. Tinti et al. reported that a Pro, Thr, Val and Phe at the -3 position was found to improve the recognition capability of this antibody but like α-PY100 also the 4G10 phosphotyrosine antibody is negatively affected by the presence of negative charge at the -1 position [46]. Re-evaluation of the obtained results in the respective studies by using anti-PY20 or anti-PY99 might thus further enhance the gained information on tyrosine phosphorylation. A recent report of Zhang et al. [66] indicated the specific changes in tyrosine phosphorylation mediated by a single A/T polymorphism of the EPIYA-B motif in Western *H. pylori* strains. This further documents the importance of knowledge about the recognition capabilities by different commerical phosphotyrosine antibodies.

Investigation of the binding specificity of α-phosphotyrosine antibodies allows valuable insights in *H. pylori*-mediated tyrosine phosphorylation events. For example, by analyzing lysates of infected cells first conclusions can be drawn [95]. However, by using this approach some drawbacks have to be considered because increasing phospho-CagA signal intensities on conventional one-dimensional gels cannot be further distinguished. Such intensification of signals might arise over time due to increased amounts of translocated CagA molecules undergoing phosphorylation at a specific site, from increased phosphorylation of multiple sites per CagA molecule, or both. Recently, we demonstrated by two dimensional electrophoresis that during infection CagA can be simultaneously phosphorylated either on one or two EPIYAs per molecule [45]. It appears that the presence of multiple differentially phosphorylated CagA protein species in host cells result in different CagA signaling involving various host binding partners, each with possible different function [45]. To clarify this issue, the generation of phospho-specific α-CagA antibodies for each EPIYA motif has to be considered as such antibodies are currently not commercially available. Until now, only little information is available about phospho-specific α-CagA antibodies [11, 25, 26, 96], however, some of them lack sufficient controls to allow clear conclusions. Thus, it remains to be indispensable to generate more reliable EPIYA-site specific phospho-antibodies to improve and augment the current understanding on tyrosine phosphorylation.

## Conclusions

Previously, we focused on the EPIYA-motifs A, B and C of Western *H. pylori* strains [48]. In the current study, we could further broaden and intensify our knowledge by addressing also the East Asian-type *H. pylori* strains. These strains carry the more potent 11-mer EPIYA-D sequence (SPEPIYATIDF) which is similar to the Western EPIYA-C sequence (SPEPIYATIDD) [45]. In Western blot experiments utilizing the α-PY99 antibody, we were able to show that the c-Src kinase is only able to phosphorylate the CagA EPIYA-C and EPIYA-D motif [45]. By comparing the results of phosphorylation of the Western EPIYA-C motif and the Asian EPIYA-D motif it becomes evident that, as expected, all antibodies able to recognize the phospho-EPIYA-C motif were able to recognize EPIYA-D motifs almost to a similar extent. For future studies, the phosphopeptide microarray technology should be considered to identify all known individual phospho-EPIYA-motifs and associated amino acid polymorphisms as was done already for human proteins [46]. In this context also antibody recognition and host effector protein binding should be included to further verify these findings. The role of single EPIYA-motifs of CagA might assist in risk predictions and improvement of the treatment of patients carrying gastric diseases. In the upcoming years, research should also focus on other bacterial effector proteins that, similar to EPIYA phosphorylation by *H. pylori*, may have impact on downstream signaling events and disease progression. This includes additional bacterial species such as EPEC, *Chlamydia*, *Bartonella, Anaplasma, Haemophilus* and *Ehrlichia* species already found to similarly play roles in tyrosine phosphorylation [29, 75–82].

## Abbreviations

BY kinases: *B*acterial t*Y*rosine kinases
*cag*PAI: Cytotoxin-associated genes pathogenicity island
EPEC: Enteropathogenic *Escherichia coli*
FBS: Fetal bovine serum
MALT: Mucosa-associated lymphoid tissue
MOI: Multiplicity of infection
PBS: Phosphate-buffered saline
PTB: Phosphotyrosine binding
PVDF: Polyvinylidene fluoride
SH2: Src homology 2
T4SS: Type IV secretion system
